# Primary obsessive slowness in a young woman who benefited from continuous psychoeducation and modeling with video recordings during hospitalization: a case report

**DOI:** 10.1186/s12888-024-05793-1

**Published:** 2024-05-21

**Authors:** Takahiko Inagaki, Daisuke Funada, Fumi Imamura, Yasue Mitamura, Yuichi Murata, Naoki Yoshimura, Shinsuke Kito

**Affiliations:** 1 Department of Psychiatry, National Center Hospital, National Center of Neurology and Psychiatry, 4-1-1 Ogawa-Higashi, Kodaira, Tokyo, 187-8551 Japan; 2 Department of Clinical Psychology, National Center of Neurology and Psychiatry, National Center Hospital, Tokyo, Japan; 3 National Center for Cognitive Behavior Therapy and Research, National Center of Neurology and Psychiatry, Tokyo, Japan; 4 Department of Psychiatric Rehabilitation, National Center of Neurology and Psychiatry, National Center Hospital, Tokyo, Japan; 5 Department of Psychiatry, Kyoto University Graduate School of Medicine, Kyoto, Japan

**Keywords:** Obsessive-compulsive disorder, Primary obsessive slowness, Psychotherapy, Psychoeducation, Modeling

## Abstract

**Background:**

Obsessive slowness, a symptom of obsessive-compulsive disorder (OCD), is characterized by compulsive behavior and significant slowness of movement. Primary obsessive slowness (POS) is defined as a condition in which a series of actions are segmented, and the patient spends an unlimited amount of time performing each action while checking each action, resulting in cessation or slowness of movement. It is often difficult to treat POS with exposure and response prevention, which is considered effective in general OCD, and no treatment has been established. Here, we discuss the effectiveness of psychoeducation and modeling using video recordings in the treatment of POS.

**Case presentation:**

We report a case of POS in a 19-year-old woman. Each action was subdivided and ordered, and the patient could not proceed to the next action without confirming that the previous step had been performed. Therefore, she could not live her daily life independently; for instance, toileting and bathing required more than 1 h, even with assistance. After more than 5 months of long-term treatment, including pharmacotherapy, psychoeducation, and modeling with video recordings, she recovered to live her daily life independently.

**Conclusion:**

Psychoeducation and behavioral therapy can effectively treat POS. Particularly, modeling with video recordings would be an easy-to-use option for POS treatment.

## Background

Obsessive slowness (OS), a symptom of obsessive-compulsive disorder (OCD), is characterized by compulsive behavior and significant slowness of movement. It has been suggested that OS is associated with increased severity of aggression, contamination, symmetry, and hoarding of OCD [1]. Primary obsessive slowness (POS)is also a symptom of OCD. Unlike typical OS, POS is defined as a condition in which a series of actions are segmented, and the patient spends an unlimited amount of time performing each action while checking each action, resulting in cessation or slowness of movement [2]. In addition, unlike most cases of OS, obsessions are ambiguous, and the mode of experience, that is, the generation of anxiety or its alleviation through compulsive acts, is unclear. Therefore, while exposure and response prevention (ERP) is considered useful for normal OCD, it is often difficult to treat POS with ERP. Instead, behavior shaping through modeling, prompting, shaping, pacing, etc., are mentioned as treatment options. However, their therapeutic effects are difficult to generalize, and response to treatment is poor [3].

Despite these characteristics that distinguish POS from general OCD, POS is viewed negatively. Ganos et al. pointed out that the slowness seen in OCD may be due to comorbid conditions, such as catatonia, depression, and parkinsonism [4]. While affirming the existence of obsessive-compulsive slowness, Veale is negative about POS, stating that slowness is only a secondary consequence of other obsessive-compulsive symptoms [5].

Although POS cannot be considered as an independent disease concept, the slowness of movement is not secondary to typical compulsive washing or repeated checking. It is also true that there are cases, such as this case we report here, where the person’s movements become slow because they subdivide their actions into smaller parts and check each action in their thoughts. At the same time, there are ambiguous but obsessive thoughts and a pattern of thought and behavior in which people carry out compulsive acts to eliminate them. This obsession is the thought that it is necessary to subdivide the action into smaller parts and confirm how the action was carried out. It is not the thought of repeating the same checking. Such pattern of thought and behavior supports the validity of considering POS as a symptom of OCD. It could not be explained by catatonia, depression, and parkinsonism.

Here, we report a case of POS in a young woman whose symptoms improved after psychotherapy during hospitalization and discuss the heterogeneity of POS and effectiveness of psychotherapy in its treatment.

## Case presentation

The patient was a 19-year-old woman born with normal development. She achieved good grades and graduated from elementary school. Her relationships with family and friends were good. At the age of 13 years, she developed obsessive compulsive symptoms, could not go outside, and dropped out of middle school because of worsening symptoms. The following year, she visited a psychiatric hospital and was diagnosed with OCD. After repeated hospitalization, she attended psychiatric daycare programs on her own for several months. However, in year X-1, her slowness relapsed, and she could not live her daily life without help. She was admitted to our hospital in months Y and X.

When admitted to our hospital, she moved extremely slowly and required several hours to eat and use the toilet. Each action was subdivided and ordered such that she was unable to move on to the next action without confirming the action she had performed. When she was unable to carry out such actions as planned, a sense of incompleteness manifested. Such compulsive behaviors were present every day for more than 2 weeks and unrelated to depression, schizophrenia, or Tourette syndrome; therefore, we diagnosed her with OCD. Because she experienced no compulsion for washing, fear of filth, or repetition of the act of checking, we ruled out the possibility of slowing down as a secondary result of these factors. We also interviewed the patient and ruled out any developmental disorders. No history of psychoactive substance or alcohol use ruled out substance use disorders. Her slowness was not based on biased values or perfectionism; therefore, personality disorder was excluded. She experienced no symptoms of parkinsonism, depressive episodes, or catatonia, and we ruled out slowness due to these pathologies. Therefore, she was diagnosed with POS. Her Yale-Brown Obsessive-Compulsive Scale (Y-BOCS) score was 28.

Psychoeducation was initiated soon after admission to our hospital and provided weekly. We asked her to review what the segmentation of action meant to her and imagine that eliminating the process would make her daily life easier. By hospitalization day 21, the patient, who had previously deemed her compulsive behavior necessary, reconsidered it to be irrational and resisted it. And then, prompting and modeling was started one month after admission. The behavioral therapy was implemented once or twice a week for about 60 min each time. Prompting and modeling for POS, in which the patient was encouraged and guided by staff and modeled to imitate the actions of others, was provided by a multidisciplinary team, including doctors, nurses, psychologists, and occupational therapists. Modeling the patient herself using videos was also effective. The video recordings were done using the patient’s mobile phone. We recorded medical staffs performing actions such as the movements of holding chopsticks and taking medication. The length of each video was approximately 1 min. Then, we made the videos available for viewing by the patient at any time. While watching a video, the patient tried to hold chopsticks and take medication in accordance with the actions of the medical staffs in the video. On hospitalization day 51, the Y-BOCS score was 31 with no improvement, although the patient could eat and take her medication with simple verbal encouragement. Toileting and bathing remained slow and required more than 1 h, even with assistance.

In order to maintain the therapeutic effect after discharge, we thought that family counseling was very significant. During admission, we also repeatedly conducted psychoeducation for the family members. In particular, we pointed out that excessive family assistance in the performance of daily personal activities deprived the patient of the opportunity to recognize the irrationality of the compulsive behavior and to feel the pain in the performance of the compulsive behavior. We explained that this would inhibit the patient’s motivation for treatments, resulting in chronicity of the obsessive-compulsive symptoms.

The medications included brexpiprazole and low-dose clomipramine before admission. The American Psychiatric Association guidelines recommend selective serotonin reuptake inhibitors (SSRIs) as the first-line medication; however, if the patient does not respond to SSRI treatment, switching to other SSRIs, clomipramine, or augmentation with an atypical antipsychotic is recommended. The patient had received multiple SSRIs (paroxetine, sertraline, and escitalopram) before admission, all of which were ineffective and was prescribed with clomipramine at a low dose. After admission, the dose of clomipramine was increased to a maximum. On hospitalization day 52, brexpiprazole was replaced with aripiprazole because of recent findings showing the efficacy of augmentation therapy with aripiprazole [6], although with off-label use. The aripiprazole dose was increased to 10 mg but worsened obsessive-compulsive slowing and was reduced to 6 mg on day 80. Memantine, which is reportedly effective [7], was also administered, and the dose was increased to 15 mg.

In month Y + 3 of the same year, the patient was discharged and continued prompting and modeling with the help of her family while visiting our hospital every week. Subsequently, the memantine dose was increased to 20 mg. Two months later, she recovered to perform toileting and bathing independently, and her Y-BOCS score improved to 20.

## Discussion and conclusions

Although POS is a symptom of obsessive-compulsive disorder (OCD), the slowness of POS differs in nature from that of secondary obsessive-compulsive slowing. We focused on the heterogeneity of POS in general OCD and investigated treatment methods that have not been established.

OCD pathophysiology is related to the dysfunction of the OCD loop (a neural circuit connecting the frontal cortex, striatum, and thalamus) [8]. The dysfunction of the OCD loop is normalized when OCD symptoms improve with pharmacotherapy or behavioral therapy [9].

Recently, differences in obsessive-compulsive symptoms in these patients have been attributed to differences in their biological bases. The biological basis of OCD abnormalities correlates with washing compulsion with the activation of the ventral medial prefrontal cortex, confirmatory acts with the activation of the basal ganglia/thalamus/dorsal frontal regions, and hoarding with the activation of the precentral gyrus/spindle gyrus/front-orbital plane [10]. However, no biological studies have targeted POS, and its biological basis remains unknown. In general, in OCD, the improvement of one obsessive-compulsive symptom spreads to other obsessive-compulsive symptoms; however, in POS, each symptom requires separate and direct treatment [2]. Another feature of POS is that patients are less likely to face maladaptive anxiety and discomfort due to a lack of irrational cognition of compulsive behaviors.

We discussed appropriate treatment for POS. First, we tried to understand the concept of compulsions and recognize the irrationality of compulsive behaviors. We talked with the patient about the activities she spent a lot of time on in her daily life and made sense of them as obsessive-compulsive behaviors. For example, when eating, the patients paused for a moment to mentally reflect on the actions they performed immediately after picking up the chopsticks. In actual psychoeducation, the psychologist talked to her about the inconvenience caused by such subdividing and confirming actions and made her imagine that eating would be easier by omitting the process. The patient gradually recognized the irrationality of the compulsion, felt discomfort, and could resist it, although only weakly.

She also worked on cognitive-behavioral therapies, such as prompting and modeling, focusing on the fact that subdividing acts and compulsive checking behaviors exhibit a certain regular pattern. Prompting and modeling are treatment methods in which the therapist encourages the patient to imitate the model behavior. When she started prompting and modeling a specific daily activity (compulsive behavior), the slowness of the activity was immediately alleviated. We hypothesized that her slow movements improved immediately after the start of modeling because information from the visual cortex was transmitted to the premotor cortex without going through the OCD loop [11]. To maintain this effect, the modeling process had to be repeated. However, because many of her daily activities were associated with compulsive behavior, the prompting or modeling of those activities was impossible to repeat. Therefore, we hypothesized that modeling using video recordings of daily activities would be visually interventional, easy to repeat, and effective. Using video recordings allowed the patient to continue modeling easily, even at home after discharge, which greatly contributed to living her own life. Through this case, we showed that psychoeducation and behavioral therapy could effectively treat POS. In particular, modeling using video recordings may be an easy-to-use option for POS treatment.

Although it is not possible to clarify the contribution of each treatment method, psychoeducation, pharmacotherapy and behavioral therapy, to the therapeutic effect, the symptoms of POS began to decrease even before the introduction of aripiprazole and memantine. In other words, it is clear that psychoeducation and behavioral therapy had a therapeutic effect.

However, there are a few limitations in this report. As this is a case report and there is no control group, the reproducibility of the treatment effects cannot be guaranteed. Furthermore, as this study did not evaluate changes in biological functions in the brain, it is unclear what mechanism is behind the improvement in clinical symptoms by the treatments. In the future, we hope that larger-scale studies with control groups will demonstrate the effectiveness of psychoeducation and modeling with video recordings in POS. Furthermore, examining the biological characteristics of POS may also demonstrate the mechanism of actions of treatments and the validity of treating POS as OCD.

## Data Availability

Not Applicable.

## Abbreviations

OCD: obsessive-compulsive disorder
POS: primary obsessive slowness
OS: obsessive slowness
ERP: exposure and response prevention
Y-BOCS: Yale-Brown Obsessive-Compulsive Scale
SSRI: selective serotonin reuptake inhibitor
